# A Location-Aware Waypoint-Based Routing Protocol for Airborne DTNs in Search and Rescue Scenarios

**DOI:** 10.3390/s18113758

**Published:** 2018-11-03

**Authors:** Armir Bujari, Carlos T. Calafate, Juan-Carlos Cano, Pietro Manzoni, Claudio E. Palazzi, Daniele Ronzani

**Affiliations:** 1 Department of Mathematics, University of Padua, 35121 Padua, Italy; abujari@math.unipd.it (A.B.); cpalazzi@math.unipd.it (C.E.P.); 2 Departamento de Informática de Sistemas y Computadores (DISCA), Universitat Politècnica de València, 46022 Valencia, Spain; calafate@disca.upv.es (C.T.C.); jucano@disca.upv.es (J.-C.C.); pmanzoni@disca.upv.es (P.M.)

**Keywords:** DTN, routing, UAVs, search and rescue

## Abstract

In this paper, we propose GeoSaW, a delay-tolerant routing protocol for Airborne Networks in Search and Rescue scenarios. The protocol exploits the geographical information of UAVs to make appropriate message forwarding decisions. More precisely, the information about the future UAV’s motion path is exploited to select the best UAV carrying the message towards the destination. Simulation results show that the proposed solution outperforms the classic DTN routing protocols in terms of several performance metrics.

## 1. Introduction

*Unmanned Aerial Vehicles* (UAVs) have frequently been coupled with the *Delay Tolerant Network* (DTN) paradigm, supporting communication and service-delivery in scenarios with intermittent connectivity. In this context, the use of UAVs equipped with various technologies (GPS, cameras, sensors, etc.) has gained a lot attention in contexts, such as monitoring, surveillance and reconnaissance [1]. Indeed, a UAV’s ability to easily and quickly reach a point of interest makes them an excellent solution for diverse tasks.

In particular, the use of a swarm of UAVs to deploy a mobile ad-hoc network makes them suitable for diverse application scenarios, like disaster events, search and rescue operations, and urban monitoring, since an infrastructure-based network may be missing or damaged, being unable to work [2]. Particularly interesting are search and rescue scenarios, where the rescue team may use a swarm of UAVs to independently check the ground area of interest in order to find possible targets, following certain route paths.

The DTN paradigm is best suited for this type of scenario due to its inherent tolerance to intermittent connectivity, providing buffering and carrying data [3]. DTNs use the store-and-forward policy, where each node holds the entire message or chunks of it (bundle) to forward it to any other node at a later stage. However, many aspects in DTNs are still an open issue, like routing. Several routing protocols have been proposed to maximize the message delivery chances, increasing message replication or relying on nodes behavior prediction. Other routing techniques rely on diverse information to correctly forward the messages among the nodes in the network. Some of these are called *context-aware* protocols, since they use information about the network to take forwarding decisions, like the nodes’ location [4].

The objective of this work is to propose and analyze a routing solution for DTNs that takes into account geographic information of the nodes and, in particular, waypoints’ location. When we consider a Flying Ad-hoc Network (FANET) application, it is very common that UAVs already have a planned movement path, or that, at least, we have known target coordinates to be reached. The UAVs, through hello messages, could communicate their scheduled plan. Thanks to this information, it is possible to devise a routing protocol based on reasonable assumptions and predictions of node encounters, in order to calculate the Time To Intercept (TTI) of a certain location, which could be the base station. In essence, each node can select the best available carrier to send a message to the destination, according to the waypoints’ location.

The remainder of this paper is organized as follows. In Section 2 we discuss general background information combining FANETs and DTNs, whereas Section 3 overviews the related work. Section 4 presents the description of our protocol. In Section 5, we show the performance evaluation of our protocol, compared with several classic DTN routing protocols. Finally, conclusions are drawn in Section 6.

## 2. Background: DTNs and Issues in FANETs

In particular scenarios, infrastructure-based services access might not always be available or convenient. Examples are disaster scenarios, where the infrastructure networks could have fallen down, or in search and rescue operations that could need the deployment of a network in areas not covered by infrastructure. On the other hand, cellular networks could become a not-optimal solution for several reasons, like coverage gaps, interoperability issues between carriers, and the service model. Classic ad-hoc networks seem to be a potential solution, but in many cases the dynamic nature of such context could affect the routing performance, due to frequent link failures, delays, packet route errors, etc. [5].

An ideal solution comes through the integration of DTNs into vehicular networks, enabling and maintaining network connectivity under high delays and intermittent links. Recently, the combination of UAVs in DTNs is starting to become popular, providing a more close approximation of a realistic case, due to the high dynamicity of UAVs in terms of speed, distances and autonomy. In Ref. [6], a detailed overview of DTN technology applied to the FANET infrastructure is provided, with special focus on tactical scenarios and DoD (Department of Defence) programs. UAVs as DTN nodes used for carrying data from and to isolated buildings is considered in Ref. [7], where a smart city is the context scenario. Other works propose the implementation of an UAV architecture to carry the data within a building, creating an indoor DTN system [8].

Several application scenarios could rely on airborne devices to perform several tasks. A particularly interesting example is represented by Nowadays; Urban/environment monitoring is another example in which UAVs can perform tasks in a distributed way. Realistic and synthetic mobility models for the scenarios described above are discussed in Refs. [1,9]. Most of these applications require an already planned route schedule, where the UAVs’ path is defined in advance. This aspect can be exploited to the advantage of the routing process.

DTN routing takes place on a time-varying topology, where links come and go, sometimes predictably. Many studies focused on the fact that mobile nodes can have a fixed path scheme: buses, satellites, etc. In this case, if we assume that a node will reach a destination, a DTN routing protocol is easily designed and effective. If the nodes are numerous and with an unpredictable, randomized mobility, then a probabilistic heuristic may be adopted.

On the other hand, when we consider a FANET, the conditions are different. First of all, UAVs do not move with a fixed path scheme; this is also due to the mission dynamicity, since the tasks could change frequently. Hence, a DTN routing protocol based on a certain type of predictability is not possible. Secondly, the number of UAVs of a particular application scenario may be small, resulting in lower density as well as lower contact chances.

## 3. Related Work

We explore the past research in terms of DTN routing. A brief description of classic DTN routing protocols is provided, as well as some related work that use location information to support the packet delivering in DTNs. Next, we discuss several proposals focused on geographic information in order to help the packet delivering.

### 3.1. Classic DTN Routing Protocols

Routing protocols for DTNs are classified into two main categories: *replication-based protocols*, which ensure better delivery ratios by allowing the packets to be duplicated in the network, and *forwarding-based protocols*, which never replicate the packets. In this section we describe the most representative DTN routing protocols, which we consider for our performance evaluation.

A first example is *Epidemic* [10], a replication-based protocol, in which nodes continuously replicate and transmit the packets to newly discovered contacts. Epidemic is resource hungry, as it does not limit replications, in order to improve the chance to deliver packets. This strategy is effective when the opportunistic encounters are purely random.

The *MaxProp* protocol [11] is a flooding-based technique in which, if a contact is discovered, all the packets attempt to be replicated and transmitted to that contact. The difference with Epidemic comes in determining which packets should be transmitted first and which ones should be dropped first. In other words, MaxProp maintains an ordered-queue based on the destination of each packet, ordered by an estimated likelihood of the path.

*First Contact* [12] is a forwarding-based routing protocol in which each node transmits the current owning packet(s) to the first encountered node. The node that receives the packet(s) acts by following the same procedure, waiting for the first available contact. The process goes on until the packet(s) arrive(s) to the destination.

*Spray and Wait* [13] is a routing protocol that attempts to gain the delivery ratio benefits of replication-based routing, as well as the low resource use benefits of forwarding-based routing. Spray and Wait achieves resource efficiency by setting a strict upper bound on the number of copies per packet allowed in the network. The Spray and Wait protocol is composed of two phases: the spray phase and the wait phase. When a new packet is created in the system, a number L is attached to that packet indicating the maximum allowable copies of the packet in the network. During the spray phase, the source of the packet is responsible for *spraying*, or delivery, one copy to L distinct *relays*. When a relay receives the copy, it enters the wait phase, where the relay simply holds that particular packet until the destination is encountered directly.

### 3.2. Location-Aware DTN Work

Several works focused on location-aware architectures in order to make more efficient the considered application scenarios.

A first example is AeroRP [14], designed for airborne telemetry applications. Although not directly related to DTN but to classic ad-hoc networks, it is worth mentioning it, since the proposal checks the location and the current trajectory of nodes to take a more precise forwarding decision. AeroRP predicts the available connection with the recipient (base station) using this information, increasing the delivery ratio and reducing the overhead with respect to classic routing protocols, such as AODV and DSDV.

In Ref. [15], a protocol performance evaluation is performed considering a search and rescue scenario in the simulation configuration. Classic DTN routing protocols are evaluated for the comparison, and the authors conclude that contact opportunities represent useful information.

The authors in Ref. [16] propose an optimization for the Public Unmanned Aerial Vehicles (UAV-P) trajectory for delivery time minimization. UAV-P act as delivery devices, storing the data and delivering them to a base station, making this system a DTN. The work faces the problem by determining the optimal route that connects all the coordinates of the information sources. Our proposal is different, since we do not act on the UAVs’ trajectory; rather, we analyze the planned trajectory to choose the current best delivery UAV.

A delay tolerant application platform deployed on the Public Transportation System is proposed in Ref. [17]. The authors analyze the possibility to provide opportunistic connectivity using a carrier-based approach based on bus routes. Simulation outcome shows good performance in several scenarios, making the proposal a practicable non real-time solution. A related and more detailed work is done in Ref. [18].

Some works focus on routing strategies that merge existing ad-hoc routing protocols with DTN characteristics. It is the case of Ref. [19], which adapts the Ad hoc On Demand Distance Vector (AODV) routing protocol for the DTN architecture. Basically, a new DTN-aware routing protocol is implemented on top of the underlying and unmodified AODV.

The authors of Ref. [20] present the possibility to make the UAVs’ movements dynamic, based on the connectivity level between neighbor nodes. The objective is to change the position of UAVs in a DTN in order to establish the best communication quality in terms of signal-to-noise (SNR) ratio and packet throughput. The optimization algorithm orders the UAVs to move in a specific direction, so that the two metrics are minimized.

## 4. Protocol Design

Our proposed solution uses two information sources regarding the nodes involved: the current location and the mission planned path under the form of waypoints. The UAVs move along predetermined paths, and follow an *a priori* known schedule. This information is used by the protocol to predict the future locations of each relaying node and the time it will reach the locations (time of arrival or time of intercept), in order to transmit any message destined to a certain recipient crossing the path of the relay node. It is hence clear that a node waits for specific selected contacts and transmits the packet only to those. This aspect makes our proposal a partial replication-based strategy, like the Spray and Wait protocol. For this reason, we name our protocol *Geographic Spray and Wait* (GeoSaW).

### 4.1. Neighbor Discovery

When position-based routing protocols are considered, the general way for a node to announce its presence to the neighborhood is by periodical beacon broadcasting. Each node locally transmits in broadcast a short hello message (beacon) in order to announce its presence and position. Each node, upon receiving a beacon, stores the information of the neighbor node in a neighbor table. If a node does not receive any beacon from its neighbors within a certain time interval, the corresponding node is considered to have left the transmission range, or to be unreachable for some reason, and is deleted from the neighbor table. In Ref. [21], the authors analyze the impact of diverse beacon settings (beacon timing, node density, node speed, etc.) on the routing performance.

In our solution, in addition to the node’s position, the future path of the node should be communicated. Typically, this information is expressed under the form of a list of coordinates, which represent the waypoints. Hence, at every connection between two UAVs, they exchange their future path in order to process a possible bundle forwarding.

Differently from classic position-based routing protocols, our DTN routing protocol (and more generally, almost all the DTN routing protocols) is just partially affected by the beaconing, because of their intrinsic characteristic. Classic position-based routing protocols need the correct neighborhood information almost every time, since they totally rely on the current position of the nodes to make the forwarding decision; in this case, inaccurate and outdated neighbor information could significantly affect the correct next-hop decision process. On the other hand, our DTN routing protocol mostly relies on the future path of nodes, which does not change over time; once the first beacon is arrived, subsequent beacons are not relevant for the routing process. Hence, we should pay attention to the beacon timing regarding only the first beacon.

In this work, we do not analyze this aspect, but we intend to explore the beaconing in the future, when moving from The One simulator to *Network Simulator 3*, which reproduces more realistically the communication and protocol aspects of the network.

### 4.2. General Description

The forwarding decision on a node is taken if (a) a new message is available at the node or if (b) a new contact with another node takes place. In these cases, the node that owns the message analyzes the future movement path of the considered contact to check if it will crosses the position of the destination. If so, the node transmits the message to that contact node, otherwise, it does nothing. In the following, we detail the two specific cases, and illustrate the forwarding mechanism. For the sake of clarity we provide some notation:

- The current node that is processing the forwarding decision of a message is denoted by *c*;
- The considered message is denoted by *m*;
- The destination of *m* is denoted by $d_{m}$
- The contact that sent *m* to *c* is denoted by *p*;
- The contact that *c* is currently checking is denoted by *u*.

**(a) m is available at c.** When a new message *m* is present in *c*, it means that either *m* is created from *c* or *m* is arrived at *c* from a neighbor node *p*. *c* starts by checking all its current neighbor nodes to find any carrier *u* that will reach $d_{m}$. If *m* is created by *c*, an initial counter field called *Time To Arrival* (TTA) is set to *infinite* and stored in *m*. TTA will be used as the minimum known time *m* will take to reach $d_{m}$ being carried by the current node, in this case *c*.

**(b) a new contact u is available to c.** In this case, *c* checks all the messages in its own buffer to find any message *m* for which *u* could reach $d_{m}$. Differently from the previous case, only this new contact *u* is checked, since the other current contacts have been already processed.

### 4.3. Contact Movement Path Checking

Once node *c* is approaching to check a contact *u* for a message *m*, the protocol works as follows. As said in Section 4.1, in addition to the neighbor current position, also the planned movement route is communicated, under the form of a set of waypoints [$w_{k}$, ..., $w_{n}$]. $w_{k}$ is the waypoint from which the node *u* is currently moving, $w_{k+1}$ is the waypoint *u* is currently reaching, and $w_{n}$ is the last mission waypoint. Each waypoint is represented as coordinates (x,y).

The first action made by *c* is the analysis of [$w_{k}$, ..., $w_{n}$] to check if any segment [$w_{i}$, $w_{i+1}$], i=k,...,n−1, crosses the transmission range of $d_{m}$; if none of the segments satisfies the condition, *c* does not transmit *m* to *u*, and waits for another event (new message or contact). If a segment satisfies the condition, this means that *u* will reach $d_{m}$ in a finite time $TTA_{u}$. In this case, node *u* is called *Final Node* (FN); in our work, we assume the ideal case in which an FN will deliver a message to the destination with very high probability, avoiding delay errors, transmission failures, etc. At this point, we can define two types of protocol variants, according to two strategy aspects to pursue.

#### 4.3.1. TTA Evaluation

**Variant 1.** Node *c* calculates $TTA_{u}$. If $TTA_{u}<\infty$, *c* transmits *m* to *u*. Otherwise, *c* does nothing. In this case, *m* is forwarded to all the nodes that will reach the destination in a finite time, without comparing the time of reaching betwee nodes.

**Variant 2.** Node *c* calculates $TTA_{u}$ and compares it with the current $TTA_{m}$ value stored in *m*. If $TTA_{u}<TTA_{m}$, *u* is a new (and better than *c*) FN for *m*, and *c* transmits *m* to it, after storing the new $TTA_{u}$ into *m*’s field. Otherwise, *c* does nothing. This variant limits the number of forwardings to the sole nodes that can faster deliver *m* with respect to the current carrier node. This limiation could be useful when we have high density network, reducing the amount of traffic of Variant 1.

#### 4.3.2. Message Deletion

**Variant 1.** Once forwarded, *m* is normally deleted from *c*’s buffer. In this way, there is only one message in the entire network, similar to the First Encounter protocol.

**Variant 2.** The deletion of *m* from the buffer is avoided in order to increase the chance to find a better FN but at the cost of increasing the message replication.

#### 4.3.3. Final Node Condition Evaluation

**Figure 1:**
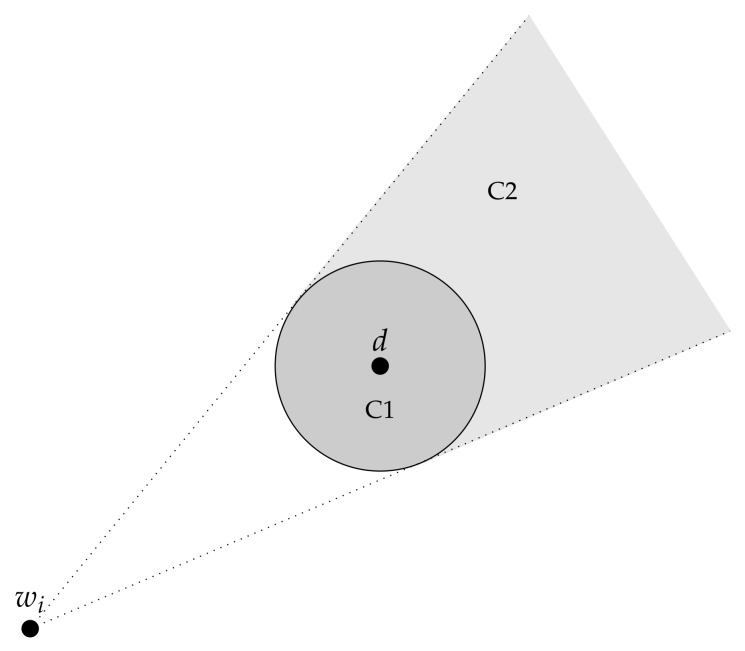
The two condition areas in which the next waypoint shall be to turn *u* into an FN.

To consider a neighbor node *u* as FN, the current node *c* evaluates *u*’s future path [$w_{k},...,w_{n}$]. For each segment [$w_{i}$, $w_{i+1}$], i=k,...,n−1, two conditions are evaluated (see Figure 1):

- **C1**: the waypoint $w_{i+1}$ is located within the transmission range of the recipient $d_{m}$.
- **C2**: the waypoint $w_{i+1}$ is located within the truncated cone projected by the transmission range circle of *d*, and whose top is $w_{i}$.

**Figure 2:**
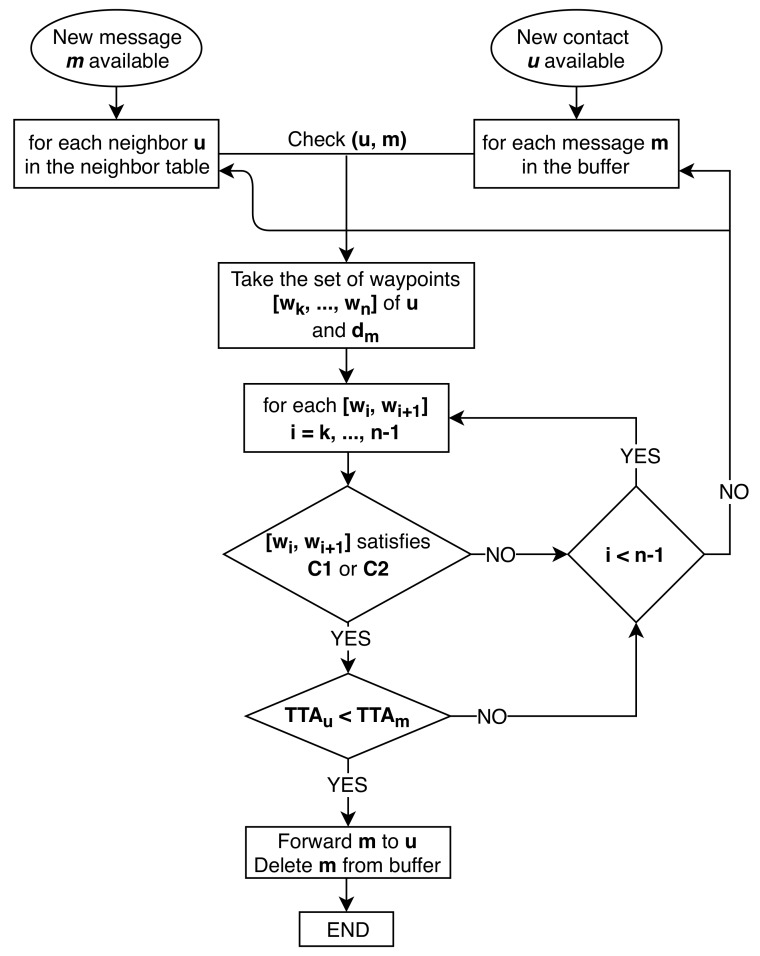
A flow chart illustration of the forwarding process of GeoSaW.

If one of these two conditions is satisfied, it is enough to make *u* an FN, as this means that this node will certainly connect to the recipient. The forwarding process is schematized in Figure 2. In particular it shows the forwarding strategy using TTA evaluation Variant 2 and Message deletion Variant 1. The chart starts with the two cases (a) and (b), which result both in checking the current *u* with the current *m*. The planned path waypoints are taken and checked with condition C1 and C2.

## 5. Performance Evaluation

In this section, we discuss the evaluation strategy of our proposal and the obtained results. For the evaluation, we use a typical search and rescue scenario where some UAVs perform a scanning search in a specific area. For each protocol configuration, we perform 100 runs so as to avoid any bias and evaluate the goodness of the following three metrics:

- Packet delivery ratio: computed as the ratio between the number of packets actually received and the number of packets sent from a source.
- Overhead ratio: the amount of extra packets needed to deliver the packet to its destination. The metric is computed as: (number of relayed packets − number of delivered packets)/number of delivered packets.
- Latency: the time taken by all packets to reach the destination.

These three metrics are studied by varying the number of UAVs, to evaluate the performance trend of the proposal under varying network density. Along with the average values, we compute the confidence interval over the 100 data samples.

To asses the protocol, we conduct two set of comparisons: The first set considers our protocol compared against the other traditional DTN routing protocols (Epidemic, Spray and Wait, First Contact, Max Prop). In particular, for Spray and Wait, the chosen number of allowed copies is set to 5. The second comparison considers different parameter combinations of the proposed protocol.

### 5.1. The One Simulation Environment

*The Opportunistic Network Environment* (The One) software is a well-know simulation tool tailored for the performance evaluation of DTN routing protocols. Written in Java, it is capable of simulating packet routing between nodes with various DTN routing algorithms and sender and receiver types. The One can generate node movements using different mobility models, and provides a graphic visualization of both mobility and packet forwarding progress through a customized interface. The software is available as open source and can be customized as needed through a main configuration file denoting the simulation components.

### 5.2. Simulation Scenario

The search and rescue scenario is a common and relevant context for UAV adoption. Trying to mimic a realistic mission objective, we took inspiration from Ref. [15] where UAVs are actively searching for a target which is located on the ground. Realistic scenarios embodying this *modus operandi* are natural disaster events (earthquake, hurricane, tsunami, etc.), where victims in dangerous or inaccessible areas need to be tracked and time is of the essence. In this context, human operators might be not capable of entering certain areas, hence employ UAVs to inspect them or UAVs are employed anyways to assist and speed up the tracking process.

**Figure 3:**
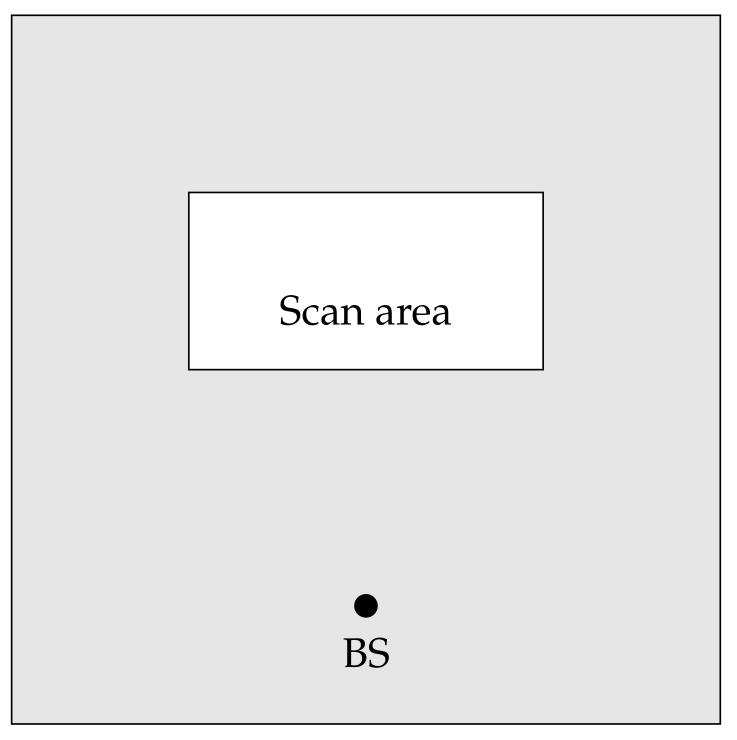
The simulated search and rescue scenario. In the white rectangle area the searching UAVs perform the search task and are confined within that area. The other nodes (delivery UAVs) are involved in other tasks, covering the larger gray area. The base station (BS) is located outside the scanning area, but within the larger area.

Under these settings, whenever a UAV has some relevant information about the search, it has to transmit the information to the base/control station, which might be located outside the search area. It might happen that no direct communication link to the base station is available and the only means to deliver the information is by relying on other nearby UAVs. On their turn, these UAVs might have different operational objectives, not necessarily related to the search operation. As an example, their focus is on a much larger operational area, containing the search one. In Figure 3 is provided a graphical representation of discussed search and rescue scenario.

Modeling this scenario, we employ the *Random Waypoint* (RWP) mobility model for the delivery UAVs. It is clear that all the waypoints of the RWP model are already planned at the start of the simulation, in order to simulate a planned path for each UAV. Communication wise, we adopt the 802.11 g standard as, with respect to other technologies, it offers an excellent tradeoff between coverage and bandwidth; in our previous test [22,23,24], we were able to communicate without packet losses up to 1.5 km. Energy wise, the electromechanical part of the drone is the most consuming one, hence employing another communication technology does not have a great impact on the final outcome. Nevertheless, one can always make use of the low power modes offered by the IEEE standard. In Table 1 are exhibited all the simulation parameters.

### 5.3. Comparison with Classic DTN Protocols

**Figure 4:**
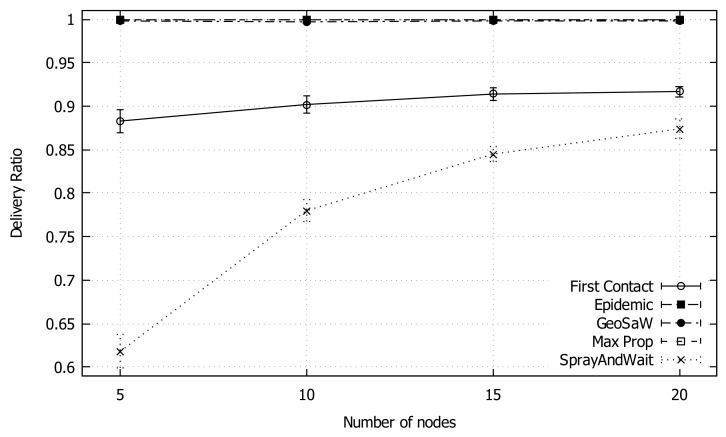
Average delivery ratio of GeoSaW compared with other DTN routing protocols when varying the number of nodes.

In this simulation, we compare our proposed protocols with the classic DTN protocols available in The One simulation environment. For this comparison, GeoSaW is configured with TTA Evaluation Variant 2 and Message Deletion Variant 1, having just one packet copy in the network. From Figure 4, we can see that the average packet delivery ratio for GeoSaW is almost 100%, comparable to Epidemic and MaxProp, under varying network density. On the contrary, the values for the First Contact protocol increase monotonically with the network density until a plateau is reached (90%), while Spray And Wait starts at a lower delivery rate, and grows as expected, although remaining under a 90% delivery rate.

**Figure 5:**
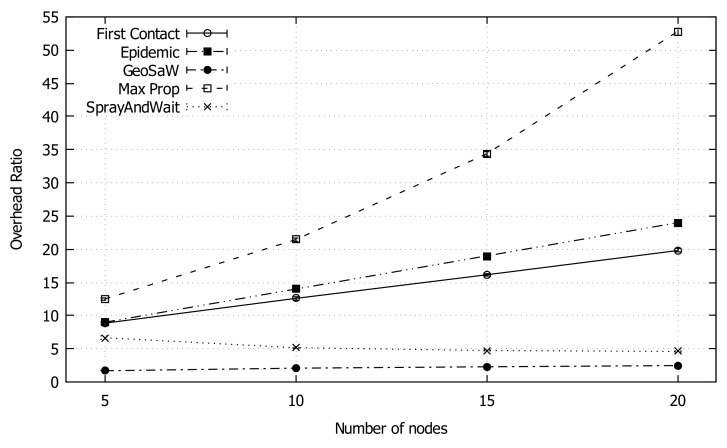
Average overhead ratio of GeoSaW compared with other DTN routing protocols when varying the number of nodes.

Figure 5 shows the average overhead as the network density increases. This value generally increases as the number of nodes increases, with the exception of Spray and Wait and GeoSaW. In fact, Spray and Wait limits the number of copies in the forwarding process, whereas in GeoSaW the number of injected packets is always one. Furthermore, although First Contact does not use a multi-copy approach, it results with more traffic overhead than GeoSaW. The other protocols do not have a packet number limitation, contributing to increasing traffic. Concluding, GeoSaW presents the best performance in terms of overhead.

**Figure 6:**
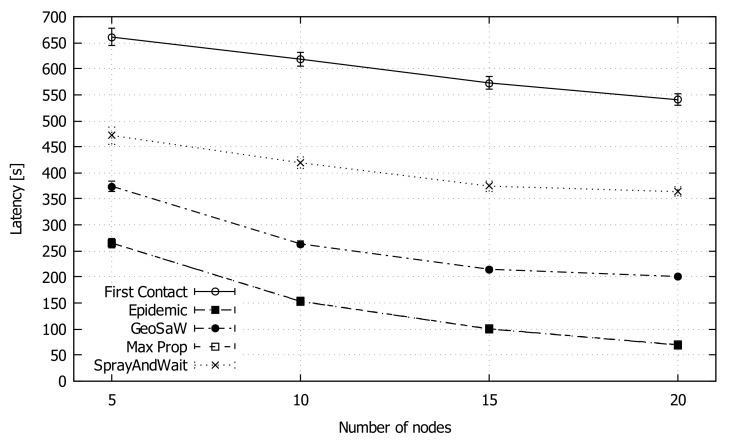
Average latency of GeoSaW compared with other DTN routing protocols when varying the number of nodes.

Figure 6 shows the average latency as the number of nodes increases. GeoSaW presents acceptable results, in particular better than Spray And Wait. The average time difference between Epidemic values and GeoSaW values is about 120 s. This difference is due to the limitation of GeoSaW path prediction: when a node meets another node, it knows the entire future path of that node, but it is not able to predict future connections with other nodes that could deliver the message faster; such ideal case could present the same performance as Epidemic in terms of latency.

### 5.4. Parameter Variation Comparison

In this simulation set, we analyze in detail our protocol by tweaking several of its configuration parameters. The first parameter is the number of allowed packet copies, ranging from 1 to 3. The second parameter taken into consideration is the permission to keep or delete the packet copy in the sending node when it is forwarded. Considering the choices, a total of 6 configurations emerge. The parameter combination used in the general comparison (previous section) is that of one copy with deletion of the packet in the sending node whenever the packet is forwarded.

**Figure 7:**
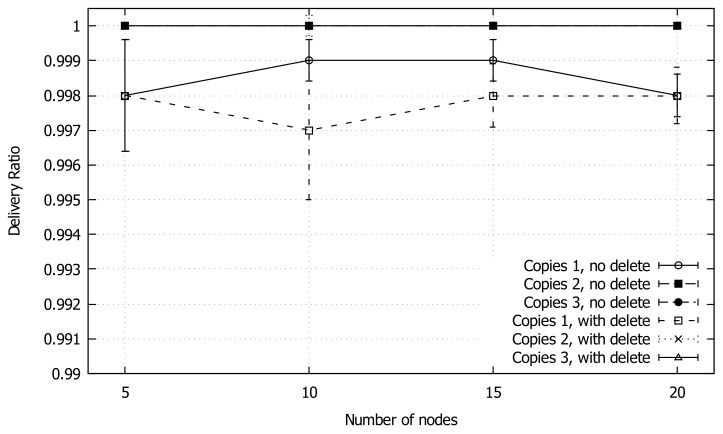
Average delivery ratio of GeoSaW with different parameters combinations when varying the number of nodes.

As illustrated in Figure 7, the delivery ratio for all the combinations is very high, with a slight decline in the case of a single packet approach. In fact, even when considering the values of the confidence intervals, the delivery ratio does not provide us with enough information about the parameter combination differences.

**Figure 8:**
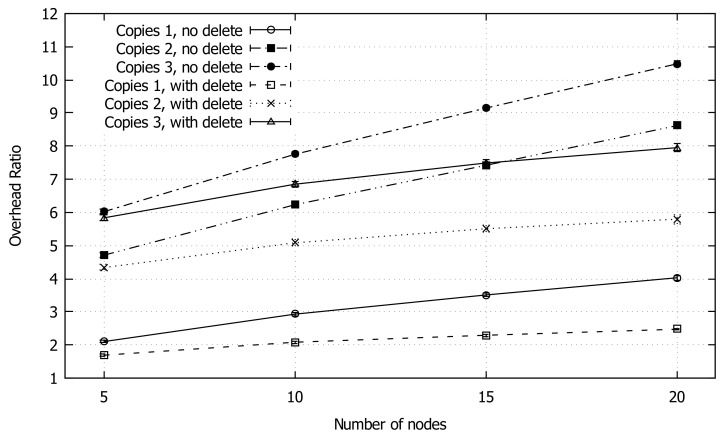
Average overhead ratio of GeoSaW with different parameters combinations when varying the number of nodes.

Figure 8 shows the overhead, which increases with the number of nodes. The one copy with packet deletion configuration performs better than the other ones, slightly exceeding an overhead of 2 in the scenarios of 15 and 20 nodes. We notice a faster overhead increase in the case of “no deletion” with respect to “deletion”; this is due to the absence of packet deletion after the forwarding, resulting in a rapid increase of packet copies in the network when increasing network density.

**Figure 9:**
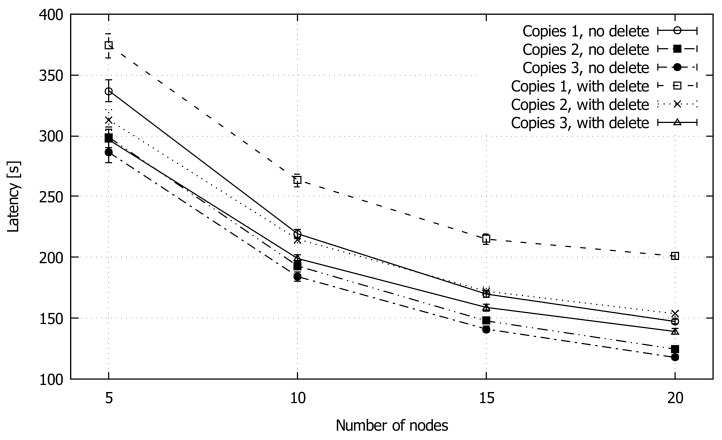
Average latency of GeoSaW with different parameters combinations when varying the number of nodes.

Finally, the packet delivery latency is shown in Figure 9. As expected, the copy with deletion combination exhibits the worst performance, while all the other combinations achieve good values, less than 150 s in the case of 20 nodes. In an operational environment, one can chose the number of copies based on the requirements of the specific application, as a tradeoff between redundancy and packet delivery delay. Another strategy worth exploiting could be that of dynamically adapting the protocol behavior whereby the number of replicas is computed dynamically based on some time to destination criteria.

## 6. Conclusions

In this work, we propose GeoSaw, a DTN routing protocol that relies on the planned UAV’s path to make the message forwarding decision. We analyzed its performance under varying scenarios, showing the advantages in terms of delivery ratio and overhead. However, the protocol still suffers from significant delays. As future work, we plan to tackle this issue on two fronts: (i) introducing an adaptable behavior to the protocol whereby the number of replicas is computed dynamically based on some time-to-destination criteria and (ii) allowing UAVs to compute and follow ad-hoc optimized path(s) that reach the recipient when necessary [16,25].

## Figures and Tables

**Table 1:** Simulation parameters.

Parameter	Value
MAC type	IEEE 802.11g, FreeSpace model
Simulation area	4000 m × 4000 m
Simulation time	60 min
Transmission range	500 m
Transmission speed	1 Mbps
Buffer size	50 MB
Message size	250 KB
Message TTL	30 min
Message creation interval	30 s
Number of scanning UAVs	5
Number of delivery UAVs	5, 10, 15, 20
Scanning UAVs mobility model	Scan
Delivery UAVs mobility model	RWP
